# High speed and adaptable error correction for megabit/s rate quantum key distribution

**DOI:** 10.1038/srep07275

**Published:** 2014-12-02

**Authors:** A. R. Dixon, H. Sato

**Affiliations:** 1 Corporate Research and Development Centre, Toshiba Corporation; 2 Komukai Toshiba-cho, Saiwai-ku, Kawasaki-shi 212-8582, Japan

## Abstract

Quantum Key Distribution is moving from its theoretical foundation of unconditional security to rapidly approaching real world installations. A significant part of this move is the orders of magnitude increases in the rate at which secure key bits are distributed. However, these advances have mostly been confined to the physical hardware stage of QKD, with software post-processing often being unable to support the high raw bit rates. In a complete implementation this leads to a bottleneck limiting the final secure key rate of the system unnecessarily. Here we report details of equally high rate error correction which is further adaptable to maximise the secure key rate under a range of different operating conditions. The error correction is implemented both in CPU and GPU using a bi-directional LDPC approach and can provide 90–94% of the ideal secure key rate over all fibre distances from 0–80 km.

Quantum Key Distribution (QKD) aims to create and provide secure key data to users for cryptographic tasks^1^. With the security based upon physical principles it is able to provide a theoretical guarantee that the keys are unknown to any third party with a high and quantifiable probability^2^, something not possible with other existing types of key distribution. From the theoretical beginnings rapid experimental progress has been made, with recent experiments demonstrating high rates of key distribution^3^,^4^,^5^ combined with wavelength division multiplexing^6^,^7^,^8^,^9^ and network operation^10^.

The procedure for generating a key using QKD divides into two distinct parts. In the first stage hardware is used for the transmission and detection of quantum states, while in the second stage the information recorded from those quantum states is post-processed using software into a final secure key. The first stage is typically where most of the research effort into QKD has been concentrated, with systems now able to operate stably and continuously at Mbit/s hardware key rates^11^,^12^. However often the second stage is neglected, especially in high key rate experiments, with secure key rates instead estimated. For a complete high speed QKD system it is also necessary for the second stage, software post processing, to be also able to operate at Mbit/s rates to avoid limiting the final secure key rate. A noteable exception to this is in Continuous Variable (CV) QKD, where the particularly challenging post-processing required has seen significant research resulting in increases in the secure rate and distance^13^,^14^. The focus of this paper however is the more commonly implemented Discrete Variable (DV) QKD.

The post-processing divides into three main steps; sifting, error correction and privacy amplification. Sifting is computationally straightforward, consisting mainly of simple bit comparison operations, and can generally be performed at high speed without much difficulty. Privacy amplification is in principle a relatively straightforward matrix multiplication operation, however in order to reduce statistical finite data size effects very large (approximately 1 to 100 Mbit per dimension) matrix sizes should be used. Computing such large multiplication quickly is a challenge, with approaches using more complex algorithms such as number theoretic transforms suggested to enable operation at both large block sizes and with short computation times^15^. Here we focus on error correction which is generally a relatively computationally complex operation. As noted recently^16^,^17^ much QKD error correction research has aimed at minimising the extra redundant information sent to correct the errors whereas in practice other parameters are also important. As the redundant information can also be intercepted by an eavesdropper and so must be removed from the secure key it is important to minimise it, but in a complete QKD system it is necessary to also consider two other parameters of the error correction; the bit throughput rate and the error correction failure rate. The throughput rate determines how much information can be processed by the error correction per time period and so sets a hard upper limit on the secure key rate. The failure rate indicates the fraction of data which cannot be corrected and so must be discarded, causing a corresponding percentage decrease in the secure key rate. For a given QKD system and operating distance it is important to find the optimum combination of all three parameters to generate a secure key at the highest rate possible.

The new contribution detailed in this work is an approach to error correction designed with this in mind, to give the highest possible secure key rate in a practical QKD system. The error correction is based on rate adaptable LDPC codes and two implementations are described, the first entirely software (CPU) based and the second using additional graphical processing unit (GPU) hardware to accelerate the throughput rate. A bi-directional approach is implemented which can increase the throughput by up to a factor of 2 for any LDPC implementation. The error correction implementation is applied to a high speed QKD system to determine and optimise the maximum secure key rate at different operating points.

## Results

We selected Low Density Parity Check (LDPC) codes^18^,^19^ for QKD error correction due to several factors. Firstly they have a range of well studied decoding algorithms available offering different compromises between computational complexity (and so data throughput rates) and failure probability. Algorithms can also be selected which perform well while disclosing close to the theoretical minimum amount of redundant information. Their flexibility allows the same error correction scheme to be employed while dynamically changing priority between throughput and information disclosure as required. Using rate adaptation techniques the codes are also able to correct efficiently over a wide range of error rates. LDPC codes further have very low communication complexity, requiring only a single message in one direction. This makes them relatively immune to the effects of network latency and also more suitable for parallel hardware implementation, where multiple units each with interactive bi-directional communication can saturate data communication channels.

LDPC is asymmetrical – one party (the encoder) calculates syndrome information from their sifted key data and provides this syndrome information to the second party (the decoder). The syndrome consists of information derived from the encoder's key data, for example by calculating the parity of subsets of the key data bits. The decoder then uses this syndrome information to correct their sifted key data so that it identically matches the encoder's. The decoder is significantly more computationally complex than the encoder, which only has to calculate the syndrome message consisting of a single iteration of parity (XOR) operations, and as such the decoder is generally the most important operation to optimise.

Two implementations of LDPC were investigated, the first based entirely in software and carrying out the decoding on a standard server PC (Intel X5675 3 GHz CPU, 3 GB RAM) and the second using a PC with an additional graphical processing unit (NVidia M2090 GPU) to perform the decoding. The decoder is belief propagation based and uses the Sum-Product algorithm^19^ with log likelihood ratio (LLR) messages. Other algorithms, including offset and normalised Min-Sum^20^, were tested and while the Sum-Product algorithm is the most computationally complex this results in it decoding successfully with the least additional information. Furthermore by implementing syndrome checking after each iteration to end decoding once all errors are corrected, Sum-Product has the flexibility to operate at the higher throughput rates of simpler algorithms by increasing the amount of additional information and so reducing the number of iterations until error corrections completes. If syndrome checks fail after a specified maximum number of iterations (typically between 50 and 100, set based on the failure rate) is reached then decoding ends and failure is declared.

Serial message update scheduling^21^ is applied on the CPU and flood scheduling on the GPU. While serial scheduling is more effective (typically reducing the number of iterations by half) the memory access model of the GPU results in better performance with flood scheduling. Both floating point (32 bit) and integer (32 bit) representations of the messages were implemented; however on the hardware tested performance was almost identical with either implementation. Using shorter bit length representations also did not improve performance. The most computationally expensive maths expression calls are evaluated using pre-built lookup tables on the CPU and native function calls on the GPU. The decoding algorithm is parallelised to run in multiple threads – for the CPU implementation 6 cores were used and the best performance was found when using one thread per logical core supported by the CPU architecture. Additionally in order to optimise the memory access pattern for the GPU implementation multiple sifted key file blocks are decoded simultaneously. Due to the use of rate adaptation (discussed below) it is not necessary for these sifted key blocks to all have the same QBER.

**Figure 1:**
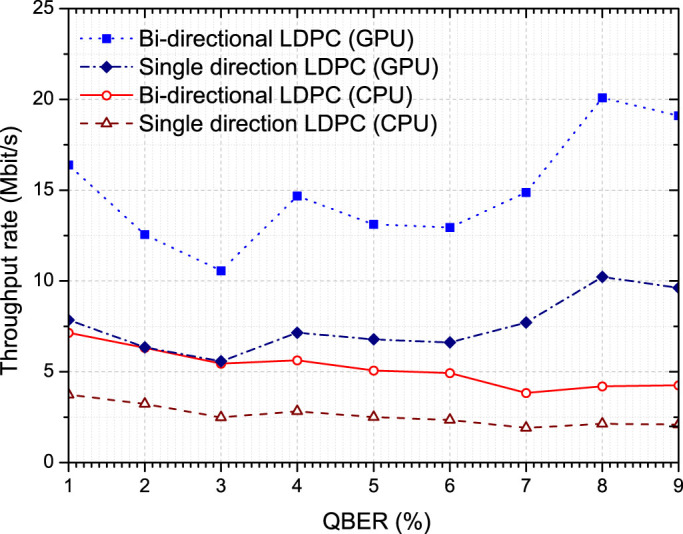
Throughput bit rate of single direction and bi-directional LDPC error correction implemented on CPU and GPU, as a function of QBER.

As mentioned above, LDPC is asymmetric in the sense the decoder is much more computationally complex than the encoder. As such the QKD transmitter's computer, assuming they perform the encoding, is idle for much of the time while the overall error correcting bit rate throughput is determined by the speed of the decoder processing on the QKD receiver's computer. To avoid this asymmetrical and inefficient use of computational resources we implement a bi-directional approach for the error correction as follows. The parties each split their sifted key data into two equal subsets, with the transmitter performing encoding on one subset (A) and the receiver performing encoding on the other subset (B) in parallel. Following encoding calculated syndrome A is sent from the transmitter to the receiver and syndrome B from the receiver to the transmitter. Decoding is then also performed by both parties in parallel, with the transmitter decoding subset B and receiver decoding subset A. As the computationally expensive decoding step is now shared equally by both transmitter and receiver then ideally (assuming the computational time for the encoding calculation is small) the error correction throughput rate should be increased by a factor of 2. Implementing this experimentally we find the throughput is increased by almost exactly this factor for both the CPU and GPU implementations, as illustrated in Figure 1. This graph shows the throughput measured as a function of error rate for both of the implementations (CPU and GPU), and also for both single and bi-directional approaches for each. In the conventional single direction mode one computer performs encoding only and the second computer decoding only, whereas in the bi-directional case both computers perform encoding and decoding in parallel.

Discrete Value QKD transmission after basis sifting is analogous to transmission through a binary symmetric channel – a classical bit flip channel where bits are flipped with a probability of *e*, with *e* the quantum bit error rate (QBER). For this class of channel the minimum amount of extra information required to correct the errors reliably is given by information theory as *H*(*e*), with *H* the binary entropy function^22^. The amount of information disclosed by real error correction is then expressible as *fH*(*e*) using an efficiency parameter *f* ≥ 1.

For LDPC the amount of information disclosed, and so the efficiency *f*, is determined by the code rate. The size of the syndrome message (*S*, the disclosed information) is related to the rate (*R*) and the size of the data to be error corrected (*N*) through *S* = *N*(1–*R*). Ten different LDPC codes were constructed with a fixed code rate between 0.55 and 0.85 and a length of 10^5^ bits using a modified progressive edge growth (PEG) algorithm^23^. Rate adaptation using puncturing and shortening is also applied to allow the effective code rate to be adjusted continuously between these fixed points^24^. Further details on the code construction and rate adaptation are contained in the Methods section. The choice of effective code rate to use (the rate of the base code and the amount of punctured or shortened bits used for rate adaptation) at each QBER is a crucial parameter in determining the overall throughput and efficiency of the error correction, and thus the final secure key rate of QKD. At a given QBER using a higher rate code will disclose less information resulting in a better efficiency and a lower rate code will finish correcting errors after fewer iterations resulting in higher throughput. Instead of fixing this choice we allow it to be dynamically adjusted by the software, parameterised by a value *p*. *p* = 0 corresponds to using the highest effective code rate which will reliably correct at each QBER, with larger values of *p* indicating lower effective code rate choices.

**Figure 2:**
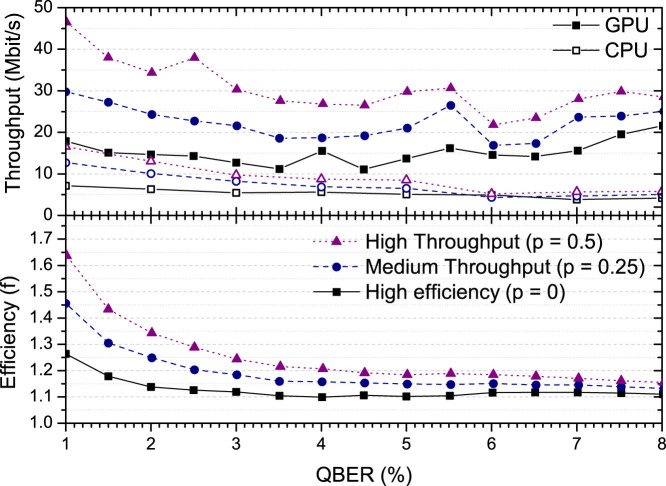
Throughput (upper panel) and efficiency (lower panel) as a function of quantum bit error rate for CPU and GPU implementations (note efficiency is the same for both CPU and GPU for a given *p*). The different lines show varying trade-offs between throughput and efficiency, parameterised by *p*.

Figure 2 illustrates the throughput and efficiency combinations for different values of *p* from 0 to 0.5. *p* can be increased further until the throughput saturates at 120 Mbit/s at an efficiency of around *f* = 2.0. At this point the number of iterations is very small, less than 5, and so further increased in throughput would only be possible by changing to a less complex algorithm such as Min-Sum.

**Figure 3:**
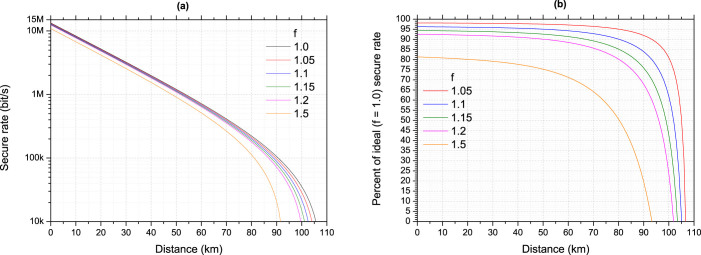
Secure key rate calculated for a high speed QKD system using ideal and theoretical error correction. Different lines correspond to different error correction efficiency (f) values. (a) The rate as a function of distance. (b) The secure key rate as a percentage of the rate obtained using perfect error correction (*f* = 1.0, black line in (a)).

Using these results we can then proceed to optimise the secure key rate of a recent high speed QKD system^25^. This system uses the efficient BB84 protocol^26^ with decoy states and its security analysis includes finite key effects and composable security (with a failure parameter of ε = 10^−10^). Its raw and secure key rate is representative of state of the art experimental QKD systems. Further details of the QKD system are provided in the Methods section. We first calculate the secure key rate using ideal error correction – that is error correction which has unlimited throughput, 0% failure rate and discloses the theoretical minimum amount of redundant information – as a benchmark. Figure 3(a) shows the secure key rate calculated for the high speed QKD system using both ideal error correction and theoretical error correction which discloses progressively more information from *f* = 1.05 to 1.5. The secure key rate as a percentage of the ideal error correction secure key rate is shown in Figure 3(b), where the effect of using imperfect error correction on the final key rate can be more easily seen. The transmission fibre is standard telecom fibre with an attenuation of 0.2 dB/km, and no implementation based bottlenecks, for example privacy amplification throughput, are considered.

**Figure 4:**
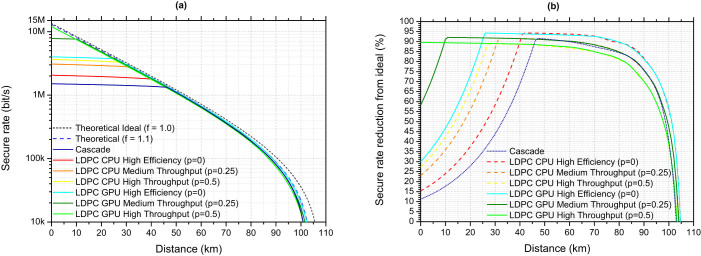
Secure key rate calculated for a typical high speed QKD system using real error correction implementations. (a) The rate as a function of distance for CPU and GPU implementations of LDPC along with a reference Cascade implementation and theoretical ideal error correction. LDPC is shown for three values of the performance tuning parameter *p*. (b) shows the secure key rate as a percentage of the rate obtained using perfect error correction (*f* = 1.0 and infinite throughput, shown as black dashed line in (a)).

Proceeding to real error correction, Figure 4(a) shows the secure key rate calculated as a function of distance using the LDPC implementations described in this paper and also a reference Cascade^27^ implementation. The LDPC implementations are shown for the same three values of *p* shown in Figure 2. Cascade as an error correction scheme is simple to implement and widely used in QKD systems. It proceeds by dividing up the input data into blocks, calculating and comparing block parity values, and then employing interactive recursive searches to correct mismatched parities.

Figure 4(b) shows the secure key rate as a percentage of the ideal rate obtained using perfect error correction (*f* = 1.0 and infinite throughput). At short distances the effect of the error correction throughput on the secure rate can be clearly seen. As the distance becomes shorter the transmission fibre losses will decrease leading to an increased sifted, and so secure, bit rate. At a certain point however the error correction schemes are no longer able to perform error correction at the rate the sifted key bits are being produced at. At this point the output rate of the error correction becomes fixed, preventing the secure key rate from rising with increasing sifted rates as can be seen in Figure 4(a). The distance this point happens at is determined by the maximum throughput of the error correction.

At distances longer than this point, the secure key rate is determined principally by the amount of redundant information disclosed by the error correction (its efficiency). This can be seen most clearly in Figure 4(b), where the secure key rate at longer distances falls roughly along three lines. These lines correspond to different efficiency points in the throughput-efficiency trade-off, with Cascade corresponding closely to the p = 0.25 value at intermediate distances and p = 0.5 at long distances. Intuitively the secure key rate changes by approximately half the change in error correction efficiency.

The failure probability of the error correction has an effect on the secure key rate, as failed blocks must be discarded. We found the optimal failure rate across different QBERs to be 0.1% on average and always below 1%, and so the direct effect on the secure key rate is small. After 1% the failure rate increases very rapidly with QBER, and so any gain in efficiency is more than outweighed by the decrease in secure key rate from discarded blocks. If the failure probability becomes high then the throughput is reduced in two ways. Firstly the secure key rate is directly affected as data is discarded, reducing the secure rate by the failure rate. Less obviously the LDPC decoder throughput will also decrease, as it will complete the full specified maximum number of iterations before failing. For successful correction the decoder will exit at an iteration earlier than this maximum once all the errors have been corrected, increasing the throughput. Conversely if the failure probability is required to be too low there is little direct increase in secure key rate (less than 0.1% at most), while the amount of syndrome information required increases significantly thus reducing the secure key rate by a much greater amount.

## Discussion

Approaching error correction as a part of a QKD system, not a standalone problem, the important metric to look at and optimise for is the final secure key rate instead of individual error correction parameters. For high speed QKD systems with sifted key rates of multiple megabits per second error correction throughput is a serious concern. As Figure 4 shows a standard error correction scheme can reduce the secure key rate to 15% or less of its possible value at short fibre distances. At longer distances however the amount of redundant data disclosed is the more important parameter, and close to the distance limit of QKD even a few percent difference in this redundant information efficiency can have a significant effect on secure key rate. As such it is advantageous to have an error correction scheme which is flexible in terms of which parameter to prioritise for different operating conditions of a QKD system. The LDPC based scheme described allows the throughput and efficiency to be continuously adjusted to maximise the secure key rate. Figure 4 shows this for 3 discrete values of a throughput-efficiency parameter *p*. In practice however as this parameter simply sets the effective code rate to use (and the effective code rate can be adjusted continually using puncturing and shortening techniques) *p* can also be adjusted continuously. Using the GPU implementation, at 0 km *p* is set to 0.5 as this maximises the secure key rate by providing sufficient throughput. As the distance increases *p* is gradually decreased until it becomes 0.25 at around 11 km (the point just before the p = 0.25 curve begins to drop in Figure 4(b)), and then 0 at around 25 km (the similar point for the *p* = 0 curve) to maximise the secure key rate by using the highest efficiency. At distances longer than this *p* remains at 0. Using this approach the secure key rate remains close to 94% of its ideal value between 25 and 60 km and remains above 90% of its ideal value for all fibre distances between 0 and 80 km. This approach can also adapt to changing conditions at a fixed fibre distance, where environmental fluctuations tend to cause variations in detector count rates and error rates over time, to allow the secure rate to be optimised continuously based on measured experimental parameters.

In order to select the effective code rate and to initialise the decoder an estimate of the QBER of the sifted key block is required. This estimate is also required for the secure key rate calculation. To estimate this we follow the standard approach of comparing in public a small sample of the key block, which is subsequently discarded. An alternative approach is to use the exact error rate of the previously error corrected block (which can be easily computed by comparing the before and after error corrected block). The first approach introduces statistical uncertainty while the second is inaccurate if the QBER changes significantly with time. An improved idea which reduces both uncertainties is to use the received syndrome information to estimate the QBER prior to decoding^28^. The approach which provides more accurate results depends on several factors including the size of the key blocks and the time variation of the error rate. However, for the secure key rate calculation in general an estimate of several different error rates is required – the minority and majority basis, signal, decoy and vacuum QBERs. Furthermore these error rates should be estimated with well defined statistical bounds for robust finite key size handing in the security calculation. As the decoy, vacuum and minority basis sifted bits are not typically error corrected but entirely used for parameter estimation we find it more consistent to also use a similar parameter estimation method (comparing bits in public) for the error corrected bits.

The Blind protocol^29^ also approaches QKD error correction from the point of view of the overall system, considering secure key rates as the important metric. This approach uses much shorter length LDPC codes (2 × 10^3^ compared to this work's 10^5^). As discussed in Refs 9,30, which also implement similar length codes, these shorter codes offer higher throughput rates and are also more suitable in an FPGA or other hardware based implementation. However the inevitable trade-off to using these shorter length codes is a reduction in efficiency and so an overall lower secure key rate in the non-throughput limited regime as the codes require more redundant information to reliably correct, although the Blind protocol does offer an improvement on this limitation. Quasi-cyclic LDPC codes have lower memory requirements and so are also suitable for hardware based implementation, and high throughput speeds have been demonstrated with these type of codes^31^.

The Cascade protocol which is traditionally used for QKD error correction has recently been shown to be able to operate at improved efficiencies, and also at high Mbit/s throughput rates^17^,^32^, making it competitive with LDPC. However as noted by the authors Cascade remains sensitive to latency in the communication channel, which in a real network may be unpredictable. Polar codes have also been implemented for QKD applications^33^ and show good throughput and efficiency, although to achieve this very large block sizes are required.

It should be noted that in all the calculations of the secure key rate only throughput limitations arising from error correction have been considered. In practice a QKD system may also have other throughput bottlenecks, for example from the privacy amplification process (especially for larger block sizes the computational time can be long), the digitisation of detector output signals and the sifting process, which would all also artificially limit the secure rate. For privacy amplification in particular there is a similar trade off as with error correction, where high throughput rates can be obtained for small block sizes at the expense of reduced secure key rates due to finite key considerations. To maximise the secure key rate of a complete high speed QKD system it is then important to fully characterise each stage from the hardware upwards in terms of throughput limits and effect on the secure key rate. Each stage's parameter's should then be optimised taking into account not just that stage's individual output but the entire system performance.

## Methods

The high speed QKD system described in Ref 25 implements the BB84 protocol using phase coding with asymmetrical Mach Zender interferometers, 3 intensity decoy states and asymmetrical basis choices. The transmission fibre is standard single mode telecom fibre (SMF-28) with an attenuation of 0.2 dB/km at the laser wavelength of 1550 nm. Photons are detected using InGaAs APDs operated in gated mode and using a self differencing^34^ technique to enable a gating frequency of 1 GHz, equal to the transmitter's laser pulse repetition rate. The APDs operate at an efficiency of 20%, a dark count probability of 2.1 × 10^−5^ per gate and an after pulse probability of 5%. The secure key rate is calculated taking into account finite key size for a key block of 1200 seconds duration and with a composable security parameter (key failure rate) of ε = 10^−10^.

In total 10 different LDPC codes are generated with rates from R = 0.85 to 0.55. The LDPC codes are randomly generated initially using a modified progressive edge growth algorithm^23^ and stored on each computer. The standard progressive edge growth algorithm aims to create an LDPC code with a specified variable node degree (the total edges connected to a variable node) distribution and also with the largest possible cycles. Cycles are formed when a variable node is connected back to itself through a chain of check and variable nodes, and short cycles generally lead to reduced performance of codes as information does not propagate efficiently. The standard PEG algorithm aims to avoid this by placing one edge at a time and using a tree expansion from each variable node to find the check node with the largest distance. The modified PEG algorithm employs in addition a check node degree distribution to choose the check node to connect the edge to in the case two or more check nodes have the same distance from the variable node. The check and variable node degree distributions are both irregular and optimised using the density evolution algorithm to give the highest performance codes.

Rate adaptation using the puncturing and shortening techniques as described in Ref 24 is implemented to allow the code rate to by dynamically adjusted. Prior to error correction the sifted key data is padded with a certain number of random bits by the encoder. The value of these bits is either disclosed to the decoder (for shortened bits) or kept secret (for punctured bits). The decoder can then reliably fix the value of the shortened bits, which increases the algorithms ability to correct the remaining bits (and thus correcting them in fewer iterations). Conversely for punctured bits the decoder will have no a priori information on these bits, unlike the bits which come from photon detections which contain noisy information, and are therefore more difficult for the algorithm to correct. More formally the effect of this is that adding shortened bits decreases the effective code rate and adding punctured bits increases it. The optimal operating QBER point for each of the 10 different rate codes is determined based on the block failure rate, and at QBER values in between these points punctured or shortened bits are added progressively to cause a smooth change in effective code rate.

## Author Contributions

A.D. performed all work described and prepared the manuscript. H.S. guided the research.
